# Effect of pre-endoscopy intake of simethicone solution on endoscopic mucosal visibility: A single blinded, placebo control, randomized trial

**DOI:** 10.12669/pjms.36.2.1241

**Published:** 2020

**Authors:** Bader Faiyaz Zuberi, Majid Ahmed Shaikh, Faiza Sadaqat Ali, Tazeen Rasheed, Zunaira Nawaz

**Affiliations:** 1 Bader Faiyaz Zuberi, Department of Medicine, Dow University of Health Sciences, Karachi, Pakistan; 2 Majid Ahmed Shaikh, Department of Medicine, Dow University of Health Sciences, Karachi, Pakistan; 3 Faiza Sadaqat Ali, Department of Medicine, Dow University of Health Sciences, Karachi, Pakistan; 4 Tazeen Rasheed, Department of Medicine, Dow University of Health Sciences, Karachi, Pakistan; 5 Zunaira Nawaz Department of Medicine, Dow University of Health Sciences, Karachi, Pakistan

**Keywords:** Endoscopy, Oesophago-gastro-duodenoscopy, Pre-medication, Simethicone

## Abstract

**Objective:**

To determine effect of pre-endoscopy intake of simethicone solution on endoscopic mucosal visibility.

**Methodology:**

A randomized, single blinded placebo control trial was done in patients undergoing oesophago-gastro-duodenoscopy for any indication at DOTs Endoscopy Suite, CHK during the period of April to June 2019. Informed consent was taken. Patients were randomly allocated in two groups. Group-A received placebo while Group-B received Simethicone. Evaluation of mucosal visibility was assessed at 4 sites (oesophagus, fundus, antrum & duodenum) by previously validated scoring. Mean of visibility scores were compared in two groups.

**Results:**

Two hundred and forty-eight patients were inducted and randomly allocate to two groups of 124 each. Mean of total sum of scores in Group-A was 8.14 ±2.44 and that of Group-B was 5.80 ±1.75 (p<0.001). Adequate visibility in Group-A was seen in 41.1% and that in Group-B was 78.2% (p<0.001).

**Conclusion:**

Use of Simethicone significantly improves mucosal visibility during OGD.

## INTRODUCTION

Oesophago-gastro-duodenoscopy (OGD) plays a crucial in diagnosis of upper GI disorders, and great importance lies in early detection of mucosal lesions for timely diagnosing and treating the hazardous diseases including malignancies. However, presence of intraluminal bubbles and foams hamper mucosal visibility, which may cause missing of subtle mucosal lesions^1^,^2^ and increasing operator’s time of performing OGD. Simethicone (polydimethylsiloxane, plus silicon dioxide) is a tasteless, odorless, antifoaming agent which reduces surface tension, transforming small gas bubbles in larger ones which are easier to move and eliminate.^3^ It reduces gas related dyspeptic symptoms, and also has a gastroprotective effect.^4^ It is neither absorbed from gastrointestinal tract nor attached to other drugs, and rarely causes ant adverse effect,^5^ can be taken up to 900 mg/day without any systemic toxicity.^6^

It has been reported that up to 13% of malignancies are missed in index OGD that are subsequently diagnosed in repeat procedure or other investigations.^7^,^8^ In presence of high definition endoscopic system, it is important that preparation should be extremely good to have proper visualization. Despite the effect of simethicone on mucosal visibility its use in OGD is not existent in practice. Neither there is any data on its use or efficacy from our region. There is dire need to establish its efficacy in OGD in a properly conducted trial.

This single blinded randomized control trial was carried out to evaluate the effect of Simethicone solution on mucosal visibility while performing OGD, with the intention of better diagnostic yield and formulating future guidelines for the use of Simethicone prior to OGD.

## METHODS

**Fig.1:**
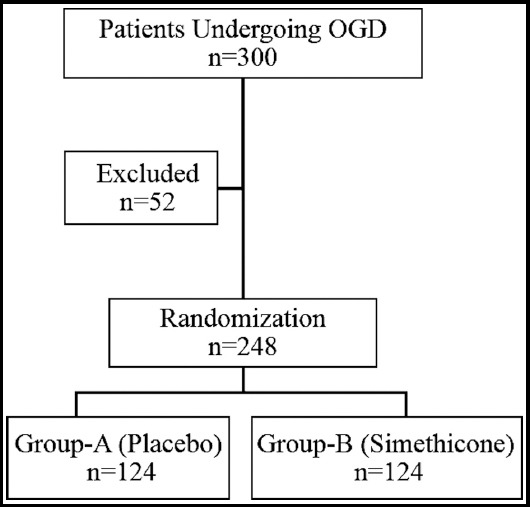
Randomization Protocol.

All patients of ages 18-80 years undergoing endoscopy for any reason at DOTs Endoscopy Suite CHK, during the period of April-June 2019 were inducted into study after taking informed consent. Study was approved by Institutional Review Board of Dow University of Health Sciences, Karachi, Pakistan vide letter # IRB-1242/DUHS/Approval/2019/57. Those having nasogastric tube insertion, stenosis of upper digestive tract, history of gastric surgery, active upper GI bleeding, cardiac and coronary artery diseases within six weeks, uncontrolled pulmonary diseases (with oxygen saturation of less than 90% at room air), pregnancy or breast feeding, thrombocytopenia (platelet less than 50,000/mm^3^), coagulopathy (INR over 1.4) and history of simethicone use within one week were excluded from study. Patients were selected using non-probability convenience sampling technique and consenting patients were randomly assigned into two groups using Random Allocation Software version 2.0. Group-A was given 50 ml of placebo while Group-B was given 15 ml Simethicone (Infacol) syrup diluted in 35 ml of water for ingestion 10 minutes before endoscopy (Fig.1). Endoscopist were blinded to the allocation status and were required to comment on mucosal visibility as per following scoring.^9^

Score 1: No foam and mucus on the mucosa

Score 2: Little foam and mucus on the mucosa without obscuring vision

Score 3: Large amount of foam and mucus on the mucosa, with less than 60 mL water to clear

Score 4: Large amount of foam and mucus on the mucosa, with more than 60 mL water to clear.

Scoring was done in four segments independently, i.e., oesophagus, fundus, antrum & duodenum. Data was collected and analysed using SPSS. Mean of scores in four segments were compared using Student’s t-test. Scores of all four segments were summed up, the minimum possible score was four and maximum possible score was 16. Total score of ≤7 was taken as adequate visibility. Proportions of patients with acceptable visibility were compared in two groups by *χ^2^*-test. Significance level was set at ≤0.05.

Sample size was calculated using previously reported visibility score of 65% and 44% in patients who received simethicone and those who did not.^10^ Calculation method used was test for two proportions using Z-test with power of 90% and alpha of 0.05. Sample size was calculated as 234 with 117 patients in each group. Sample size was calculated using PASS 2019 software using formula:

n = (Zα/2+Zβ)2 * (p1(1-p1)+p2(1-p2)) / (p1-p2)2

where Zα/2 is the critical value of the Normal distribution at α/2 (e.g. for a confidence level of 95%, α is 0.05 and the critical value is 1.96), Zβ is the critical value of the Normal distribution at β (e.g. for a power of 80%, β is 0.2 and the critical value is 0.84) and p1 and p2 are the expected sample proportions of the two groups.

## RESULTS

Three hundred patients were initially selected for the trial, fifty-two were excluded for various reasons. Two hundred forty-eight patients fulfilling selection criteria were inducted and allocated to two groups of 124 each with randomization table created by Random Allocation Software. Group-A consisted of 76 (61.3%) males and 48 (38.7%) females. Group-B had 66 (53.2%) males and 58 (46.8%) females. Mean age ±SD in Group-A of males was 35.96 ±9.0 years while that of females was 34.75 ±9.12 years. There was no significant difference in age between gender in Group-A (*p*=0.47; df=122; 95% CI -2.09 to 4.5). Mean age ±SD in Group-B of males was 46.91 ±14.73 years while that of females was 46.57 ±16.31 years. There was no significant difference in age between gender in Group-B (*p*=0.903; df=122; 95% CI -5.18 to 5.86). Details are given in Table-I.

**Table-I:** Demographic details of patients.

	Group-A		Group-B	

	Male	Female	Male	Female
n (%)	76 (61.3%)	48 (38.7%)	66 (53.2%)	58 (46.8%)
Age mean ±SD (years)	35.96 ±9.0	34.75 ±9.12	46.91 ±14.73	46.57 ±16.31

Visibility Scores from different segments of upper GI tract showed no significant difference between both groups in oesophagus, but significantly better visibility scores were observed in Fundus, Antrum and Duodenum on statistical analysis using ANOVA. Details are given in Table-II.

**Table-II:** Mean visibility scores according to site and statistical analysis by ANOVA.

	Group	Mean	SD	*p*-value
Oesophagus	Group-A	1.39	0.489	0.108
	Group-B	1.29	0.456	
Fundus	Group-A	2.43	0.866	<0.001
	Group-B	1.42	0.700	
Antrum	Group-A	2.70	0.954	<0.001
	Group-B	1.63	0.749	
Duodenum	Group-A	1.62	0.694	0.037
	Group-B	1.46	0.500	
Total Score	Group-A	8.14	2.437	<0.001
	Group-B	5.80	1.748	

The scores from four segments of GI tract were summed up. The means of the total sum score were compared among groups by Student’s t-test that showed that mean score ± SD of Group-A was 8.14 ±2.44 and that of Group-B was 5.80 ±1.75 (*p* <0.001; df=246; 95% CI 1.808 to 2.869). The analysis showed significantly better mucosal visualization in Group-B.

Variable of total score was recoded if the score was ≤7 or >7 to see if the mucosal visibility score was adequate or not. The results showed that the adequate visibility in Group-A was seen in 51 (41.1%) and that in Group-B was 97 (78.2%). Statistical analysis using *χ^2^* test showed that mucosal visibility was significantly better in Group-B (*p* <0.001), details in Table-III. No adverse effects due to simethicone were reported in our study.

**Table-III:** Comparison of mean visibility scores and adequate visibility with statistical significance.

	Group-A	Group-B	P-Value	Statistical test used
Mean Visibility Scores	8.14 ±2.44	5.80 ±1.75	<0.001	Students t test
Adequate Visibility	51 (41.1%)	97 (78.2%)	<0.001	Chi-square test

## DISCUSSION

This study demonstrates improved mucosal visualization on OGD after Simethicone. The effect is highly significant as shown by statistical analysis. This improvement was also shown by Elvas L et al. in a single blinded, placebo control study on the impact of simethicone on quality of reporting, significant improvement in mucosal visualization and detection of subtle lesions like dysplasia and small polyps and changes of Barrett’s.^11^ They used three groups and used water in first group, simethicone in second and simethicone and N-acetyl cystine in the third group, where they showed significant improvement in mucosal visibility in those who received simethicone.^11^ Subtle findings are difficult to detect and could easily be missed in presence of mucous and foam.^12^ Similar results were also reported by Monrroy H et al.^10^ This is also reported that pre-procedure drink containing simethicone not only significantly improves mucosal visibility during OGD but also reduces the need for flushes during the procedure.^13^

In our study we did not come across any adverse effect in patients who received simethicone. This finding was similar to many other trials carried out to see the safety of pre-endoscopic use of simethicone.^9^ Despite these findings some centres do not advocate premedication before OGD due to several reasons; such as increase endoscopy schedule time; the worry of hypersensitivity reaction from medications (N-acetylcysteine); and the worry of aspiration from drinking larger amount of premedication just before OGD. As in Singapore where major proportion of population comprises of aging population, endoscopy is commonly carried out to assess swallowing dysfunction.^14^ They use 100 mL premedication solution along with moderate sedation before gastroscopy. This puts these patients at risk for aspiration, especially in patients with stroke.^15^ Main reasons of having no adverse effects in our study are possibly because we used very minimal amount of premedication (15 ml of simethicone in 35 ml water) and it was given 10 minutes before procedure, not just before the procedure. Hypersensitivity did not occur with simethicone which might be a fear if N-acetylcystein were have been used as premedication.

Simethicone has also been found very helpful in small bowel preparation in capsule endoscopy and there are several studies to document this effect.^16^-^18^ Furthermore multicentre randomized control trial also highlighted that the use of simethicone along with low volume of large bowel preparation solution (Clensia) before colonoscopy resulted in better tolerability by patients and almost equal mucosal visibility by colonoscpist as compared to standard 4 liter PEG solution for large bowel preparation.^19^

To date, there is no international consensus on premedication use for upper gastrointestinal endoscopy. For example, Japanese endoscopists routinely use premedication to achieve better visibility of the gastric mucosa. Contrary to that, Bhandari P, et al. in their study conducted in Japanese tertiary referral centre among 112 participants, did not recommend the routine use of mucosal clearing agents like Gascon and Pronase prior to or as targeted during gastroscopy, as they found no difference in endoscopic time, though they required fewer flushes during endoscopy.^20^ There is also no standard recommendation or guidelines for such practice in China.

As a result of the present study, we recommend the routine use of premedication with simethicone prior to OGD as it improved visibility of the mucosa and found safe. However, further studies are required to answer whether improving endoscopic visibility will result in increased detection of small but important lesions in the upper digestive tract.

### Limitation of the study

It includes use of amount of water requiring for flushing to improve mucosal visualization is operator dependent and may not be considered as an adequate measuring tool. The development of a validated and an agreed scoring tool for mucosal visibility as well as consensus regarding dose and timing of simethicone is mandatory before running further trials to validate findings of current study and previously published trials. Comparison between Simethicone versus NAC, Pronase versus NAC and Pronase versus Simethicone and may also need to be explored before the routine use of Simethicone as premedication for OGD.

## CONCLUSION

Use of Simethicone in patients undergoing OGD improves mucosal visibility during procedure and is safe. It should be recommended as standard procedure before OGD.

### Author`s Contribution:

**BFZ:** Conceived the study and gave final approval of manuscript, is responsible for integrity of research.

**MAS:** Did data collection and analysis.

**TZ:** Drafted the article.

**FSA:** Did critical revision for important intellectual content.

**ZN:** Did statistical analysis and collected study data.
